# How we remember music tempo: the role of spontaneous motor tempo in recall and preference

**DOI:** 10.3389/fpsyg.2025.1631625

**Published:** 2025-10-10

**Authors:** Kyoko Hine, Yoshitomo Wakana, Shigeki Nakauchi

**Affiliations:** Department of Computer Science and Engineering, Toyohashi University of Technology, Toyohashi, Japan

**Keywords:** recollection, spontaneous motor tempo, music, tempo, tapping

## Abstract

Musical experiences—specifically in terms of how we prefer or remember them—differ among listeners, even when we listen to the same piece of music. Recent studies have suggested that spontaneous motor tempo (SMT), which refers to the pace of repeated body movements, predicts preferred music tempo. However, the question of whether SMT is related to recalled music tempo remains unanswered. We investigated whether SMT is related to recalled music tempo. The participants in this research performed three tasks—recall (no interval), recall (8-s interval), and preference—in which they adjusted music tempos under different conditions. SMT was assessed on the basis of a finger tapping task. Linear mixed models revealed that while the original music tempo predicted adjustments across tasks, SMT significantly predicted the adjusted tempo in the recall (8-s interval) and preference tasks but not in the recall task (no interval). These results suggest that the rehearsal of music tempo may be influenced by SMT.

## 1 Introduction

Music is a universal aspect of every culture, although musical experiences differ among listeners. For example, even when individuals listen to the same piece of music, they often differ in terms of how they prefer or remember its tempo—which is defined in terms of the pace of events (McAuley, 2010; Bauer et al., 2015; Drake et al., 2000; Iwanaga, 1995; Karageorghis and Priest, 2008; Vigl et al., 2024). Preferred music tempo refers to the pace that a listener views as most enjoyable or appropriate, whereas recalled music tempo reflects the speed at which the listener remembers the music after listening. Several studies have reported a wide range of preferred music tempos (Fraisse, 1982; McAuley et al., 2006). Additionally, researchers have reported evidence concerning individual differences in recalled music tempo (Vigl et al., 2024). Understanding how these two types of tempi are produced may shed light on how preference and memory interact in the context of musical experience. However, the manner in which such tempi are produced remains poorly understood.

Recently, spontaneous motor tempo (SMT), which is defined in terms of the pace of self-paced, repetitive movements such as finger tapping, has been reported to predict preferred music tempo (Hine et al., 2022). SMT has been reported to reflect an individual’s internal timing mechanism or “internal clock” (McAuley, 2010; Repp, 2005), which plays a central role in both motor and perceptual timing tasks. In line with this view, Hine and colleagues reported that individuals who exhibit a faster SMT prefer faster music tempos than do individuals who exhibit a slower SMT. SMT involves not only finger tapping but also walking and clapping, which are known as naturally paced behaviors (e.g., Engler et al., 2024; MacDougall and Moore, 2005), thus suggesting that SMT reflects a voluntarily produced internal timing that may underlie broader temporal coordination.

There are several theoretical reasons to predict that SMT may influence not only preferred tempo but also recalled tempo. Recalling a previously heard tempo, especially following a delay, requires the internal generation and reproduction of a temporal structure without external cues. This process engages internal timing mechanisms that govern temporal estimation and reproduction. SMT, as an indicator of an individual’s internal timing tendency, may provide a reference framework for such mental reconstruction. If this is the case, individuals who exhibit faster SMT may tend to reconstruct musical tempo at a faster rate, whereas those who exhibit slower SMT may reconstruct the tempo at a slower rate. In support of this view, studies on synchronization and continuation tapping have reported that, in the absence of external pacing, the tempos produced by individuals often drift toward those individuals’ preferred tempos (Collier and Ogden, 2004; McPherson et al., 2018). Furthermore, temporal reproduction has been reported to be biased by internal priors or default timing tendencies, particularly under conditions involving increased memory demands (Jazayeri and Shadlen, 2010). These findings suggest that SMT may serve as a cognitive anchor or attractor during tempo recall, especially when memory maintenance is required. Thus, if recalled tempo indeed reflects internally guided temporal production, it is plausible to suggest that SMT plays a role similar to that observed in the context of tempo preference.

In the present study, we investigated whether recalled music tempo is related to SMT. Although SMT has been reported to predict preferred music tempo, its relationship with recalled music tempo has not yet been examined directly. To clarify this relationship, we conducted a behavioral experiment in which participants performed recall and preference tasks involving musical tempo. According to the working memory framework (Nees, 2016), the recall task without an interval relies mainly on auditory sensory memory, which briefly holds detailed timing information. In contrast, the recall task with an 8-s interval engages short-term memory with rehearsal, thereby requiring the active maintenance and reproduction of temporal information. Because SMT reflects an internal timing mechanism that may play a stronger role in the contexts of memory maintenance and rehearsal, we expected the relationship between SMT and recalled tempo to be stronger in the 8-s interval condition than in the no-interval condition. We hypothesized that individuals who exhibited faster SMT would recall faster tempos, particularly under the 8-s interval condition. SMT was also measured on the basis of a tapping task to examine the roles that it plays in both tempo preference and memory.

## 2 Method

### 2.1 Participants

We calculated the sample size required for this study with the assistance of the samplesize_mixed function in R, specifically with the sjstats package (Lüdecke and Lüdecke, 2019). At an effect size of 0.25, a power of 0.90, a significance level of 0.05, and a total of 3 cluster groups, a total sample size of 674 was calculated. Since we aimed to collect thirty data points from each participant, we recruited 23 participants (including one female and 22 males; their ages ranged from 21 to 26 years, with a mean age of 23.1 years and a standard deviation of 1.1) for this experiment. All participants were of Asian descent and had normal hearing and normal or corrected-to-normal vision. Informed consent was obtained from all participants. The experimental procedures used in this study were approved by the Committee for Human Research at Toyohashi University of Technology (approval number: 2021-2). All the experiments were conducted in accordance with the principles stipulated in the Declaration of Helsinki.

### 2.2 Music stimuli

We prepared three music lists of ten piano solo songs. The songs were downloaded from the Classical Piano Midi Page (2018) and mfiles (2018) websites. These music lists were used during the recall (no interval), recall (8-s interval) and preference tasks. The combinations of the lists and the tasks were consistent. The music lists are presented in Appendix A. When the beats contained in the musical instrument digital interface (MIDI) file were not quarter notes, the tempo of the music was calculated by using a quarter note as the beat. The average tempos for the three lists were 121.1 beats per minute (bpm, ranging from 40 to 231 bpm), 120.9 bpm (ranging from 58 to 195 bpm), and 118.7 bpm (ranging from 67 to 197 bpm). To control for potential confounders, we confirmed that the three lists were equal in terms of tempo and number of notes, as these factors could influence individuals’ preferred music tempo (Hine et al., 2022). All music was presented on the basis of the default MIDI synthesizer by Processing.

### 2.3 Procedure

**Figure 1:**
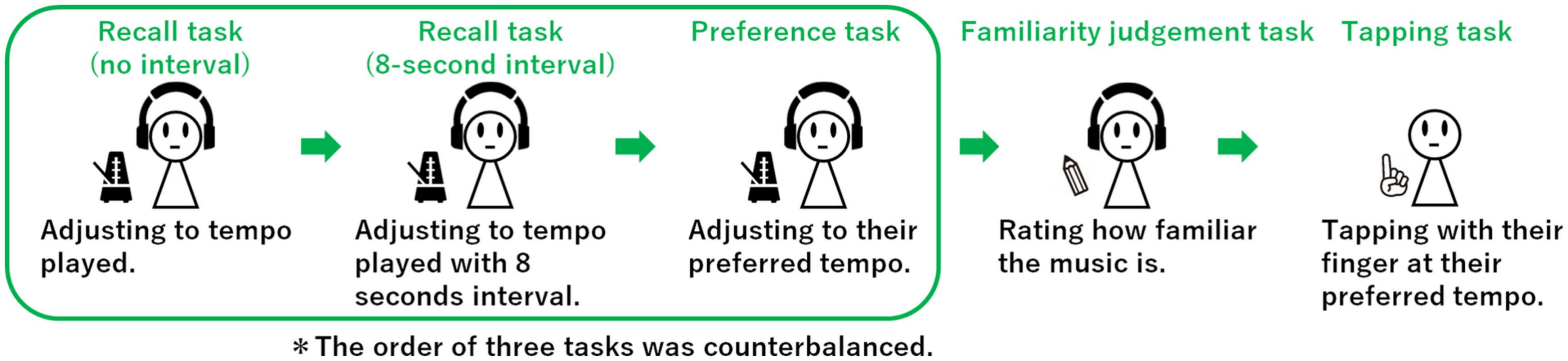
Experimental procedure. The participants engaged in the recall (no interval), recall (8-s interval) and preference tasks in a random order. The participants subsequently completed the familiarity judgement task with respect to the music used in the experiment. Finally, SMT was estimated on the basis of a tapping task.

The experiment consisted of five tasks: a recall task (no interval), a recall task (8-s interval), a preference task, a familiarity judgement task and a tapping task. The order of the recall (no interval), recall (8-s interval) and preference tasks was randomized among the participants, whereas the tapping task was always performed last (see Figure 1).

#### 2.3.1 Recall task (no interval)

In the recall task (no interval), participants were required to adjust the tempo of a piece of music to match the tempo of the piece to which they had just listened. First, the participants listened to the first 15 s of a piece of music played at its original tempo. Immediately thereafter, the same piece of music was played at 120 bpm, which was approximately the average tempo across the ten pieces of music included in the list. The participants were then asked to adjust the tempo to match the tempo that they had previously heard by pressing the corresponding key (i.e., the up or down arrow) on a keyboard. Each key press instantly changed the tempo of the music by 1 bpm. Once the participants were satisfied with the tempo, they pressed the enter key. Until the enter key was pressed, the music continued to loop. When the music was looped, a short interval was inserted, thus allowing participants to recognize that the music was being repeated. This process was repeated for each of the ten pieces of music.

#### 2.3.2 Recall task (8-s interval)

The procedure used for the memory task was the same as that employed for the recall task (no interval); however, an 8-s retention interval was included between the initial listening and tempo adjustment steps. During the 8-s retention interval, no auditory stimulus was presented. Participants were not allowed to make any movements during the interval, including tapping their hands or feet, and they were instructed to maintain the tempo mentally.

#### 2.3.3 Preference task

The procedure used for the preference task was adapted from Hine et al. (2022). First, the participants listened to the initial 15 s of a music piece that was played at 120 bpm. Subsequently, the same piece of music was presented with the same tempo (i.e., 120 bpm), and the participants adjusted the tempo to suit their preferences. Once they reached their preferred tempo, they confirmed that fact by pressing the enter key. Until the enter key was pressed, the same piece of music looped continuously. The participants adjusted the tempo of 10 different music pieces, which were presented in a random order.

#### 2.3.4 Familiarity judgement task

After the participants completed the recall (no interval), recall (8-s interval), and preference tasks, they performed a familiarity judgement task. The participants rated the familiarity of each piece of music by indicating one of three options, i.e., unfamiliar, possibly unfamiliar, or definitely familiar, regardless of whether the tempo matched their usual experience of the music in question. They provided their ratings on an answer sheet and pressed the enter key to proceed to the next music piece. The music looped continuously until the enter key was pressed. All 30 music pieces were presented in a random order.

#### 2.3.5 Tapping task

Following the familiarity judgement task, the participants completed a tapping task. They were instructed to tap the index finger of their dominant hand at a pace that they found to be natural or preferred. This tapping was performed on an iPad (Apple), which recorded the tapping speed. No visual or auditory stimuli were presented during this task. Data were collected over two 30-s trials, and participants were allowed to take as much time as they needed between trials. After the participants completed the tapping task, they were debriefed. The tapping tempo was calculated as twice the number of taps for each participant and converted to bpm for analysis. The tapping task was included at the end of the experiment to avoid potential systematic effects of tapping tempo on participants’ performance on the tempo-related tasks. Such effects would have been problematic in light of the objectives of our study.

## 3 Results

First, we summarized the familiarity ratings provided in response to the familiarity judgement task. In total, 477 pieces of music were rated as “unfamiliar,” 157 pieces of music were rated as “possible unfamiliar,” and 56 pieces of music were rated as “definitely familiar.” Familiarity with a piece of music has been reported to affect perceptions of tempo (Hine et al., 2022; Iwanaga and Tsukamoto, 1998). Thus, to determine whether familiarity ratings affected the relationship between tapping tempo and task type, we constructed a linear mixed model in which adjusted tempo served as the dependent variable and the three-way interaction (familiarity × tapping tempo × task type) served as the independent variables, whereas participant and stimulus were included as random effects. The interaction was not statistically significant, *F*(9, 260.06) = 1.62, *p* = 0.11. Based on this result, we decided to include all data in the final analyses, regardless of rated familiarity.

With respect to recall tasks, the difference between the adjusted tempo and the original tempo was calculated. The differences were 25.20 (*SD* = 23.12) in the recall task (no interval) and 19.80 (*SD* = 18.94) in the recall task (8-s interval). Also, the repeated correlation between the original tempo and the adjusted tempo was calculated. The correlations between the original tempo and the adjusted tempo were 0.81 (*p* = 0.00) for the recall task (no interval) and 0.81 (*p* = 0.00) for the recall task (8-s interval). For both recall tasks, music with faster original tempos tended to be adjusted to faster tempos, thus suggesting that participants were sensitive to tempo differences across stimuli. With regard to the tapping task, the range of tapping tempo was 62–174 bpm. The average value was 108.17, and the standard deviation was 24.93.

**Figure 2:**
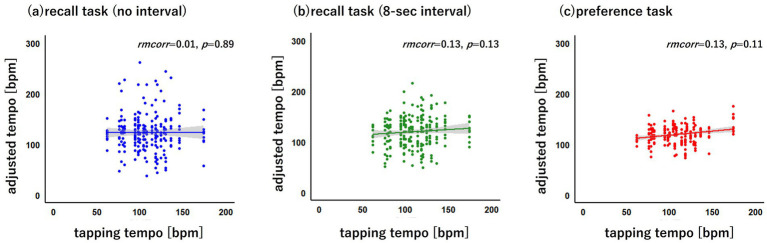
The relationships between the tapping tempo and the adjusted tempo across the recall (no interval), recall (8-s interval), and preference tasks. The X-axis represents the finger tapping tempo [bpm], and the Y-axis represents the adjusted tempo [bpm] for each task. The line represents the regression line with 95% confidence intervals.

Figure 2 illustrates the relationship between the tapping tempo and the adjusted tempo for each task. To compare the relationships between adjusted tempo and tapping tempo across the recall (no interval), recall (8-s interval) and preference tasks, a linear mixed model was developed and analysed. The model included adjusted tempo as the dependent variable, the original music tempo and the interaction between tapping tempo and the type of task as the independent variables, and the participants and stimulus as the random effects. The analysis was performed in R on the basis of the lme4 package (Bates et al., 2015), and a mixed effects model was constructed (Brown, 2021; Singmann and Kellen, 2019).

The linear mixed model analysis yielded an Akaike information criterion (AIC) of 6050.5 and a Bayesian information criterion (BIC) of 6086.8. The conditional and marginal *R*^2^ values, which were calculated with the assistance of the r2 function of the performance package in R (Nakagawa and Schielzeth, 2013), were 0.62 and 0.43, respectively. The fixed effects of the linear mixed model are presented in Table 1. The original music tempo significantly helped predict the adjusted tempo [*t*(27.966) = 8.120, *p* < 0.01, Cohen’s *f*^2^ = 0.724, power = 1.00]. With respect to the recall (8-s interval) and preference tasks, the tapping tempo significantly helped predict the adjusted tempo [*t*(43.001) = 2.029, *p* = 0.049, Cohen’s *f*^2^ = 0.025, power = 0.62; *t*(43.007) = 2.606, *p* = 0.013, Cohen’s *f*^2^ = 0.058, power = 0.55, respectively], whereas the tapping tempo did not significantly help to predict the adjusted tempo in the recall task (no interval) [*t*(43.002) = 1.598, *p* = 0.118, Cohen’s *f*^2^ = 0.000, power = 0.04]. The power was calculated on the basis of the powerSim function in the simr package in R, in which context 200 simulations were performed (Green and MacLeod, 2016).

**Table 1:** Fixed effects in the linear mixed model of adjusted music tempo.

Predictor	Estimate	SE	df	*t*	*p*	95% CI (lower)	95% CI (upper)	Significance
Intercept	62.529	7.551	44.720	8.281	0.000	49.519	75.200	***
Original music tempo	0.402	0.049	27.966	8.120	0.000	0.326	0.496	***
Recall task (no interval) and tapping tempo	0.070	0.044	43.002	1.598	0.117	0.000	0.136	
Recall task (8-s interval) and tapping tempo	0.089	0.044	43.001	2.029	0.049	0.015	0.157	*
Preference task and tapping tempo	0.114	0.044	43.008	2.606	0.013	0.043	0.187	*

All analyses were conducted with the assistance of a forced entry algorithm in R.

*p < 0.05, ***p < 0.001.

## 4 Discussion

The current study investigated whether SMT is related to recalled music tempo. Our results revealed that SMT significantly predicted the adjusted music tempo in the recall task (8-s interval) and the preference task, but it did not predict the adjusted music tempo in the recall task (no interval).

While SMT is related to the recalled music tempo when an 8-s interval is included, SMT is not related to the recalled music tempo when such an interval is not included. Nees et al. (2017) compared the accuracy of music retrieval between conditions in which articulatory rehearsal was either suppressed or not suppressed over an 8-s retention interval. That study suggested that a phonological loop was required to rehearse the tempo of a piece of music when an 8-s retention interval was included. In the current study, both recall tasks required a given music tempo to be maintained. However, a relationship with SMT was observed only in the recall task, in which context an 8-s retention interval was necessary. These results suggest that SMT, which reflects individuals’ internal clock, may be involved specifically in the rehearsal stage rather than in storage within working memory itself. In future studies, to clarify the role played by the rehearsal stage directly, it will be necessary to examine recalled music tempo under conditions with and without rehearsal suppression when the same interval is maintained.

Why could the recalled tempo with an interval of 8 s be predicted by the tapping tempo in the current study? Several studies have investigated why tempo drift occurs during isochronous tapping tasks, in which context participants are required to tap at a constant pace (Collier and Ogden, 2004; Vorberg and Wing, 1996). These studies have proposed models suggesting that such drift is caused not only by motor processes but also by the timekeeping process; they have also suggested that the drift resulting from the timekeeping process varies among different individuals. Moreover, researchers have reported that music performers exhibit individual drifts toward their preferred tapping tempo (Zamm et al., 2018). From this perspective, internal factors might explain the effect of SMT on the adjusted tempo in the recalled tempo task with an 8-s interval in the present study. In particular, McPherson et al. (2018) asked participants to perform spontaneous motor tempo tasks, which required self-paced pulse generation without external rhythmic cues, as well as synchronization–continuation tasks, which included a continuation phase without external stimuli. These authors argued that, in the absence of external pacing signals during the continuation phase, participants rely heavily on internal timing mechanisms to maintain tempo. This finding is closely in line with the results of our recall task involving an 8-s interval, in which context participants reproduced the tempo following a delay without ongoing auditory input. Such a temporal gap likely shifts temporal control from externally driven entrainment toward reliance on internal timing processes, in which context SMT serves as the individual’s internal reference for timing. Therefore, the significant prediction of adjusted tempo by SMT in the current study suggests that participants use their endogenous tempo as a cue when external information is unavailable.

The current study revealed that SMT is related to the recalled music tempo, which is during through the rehearsal stage. SMT is believed to reflect individuals’ “internal clock” (e.g., Craik and Hay, 1999). From this perspective, the rehearsal of the presented music tempo may be influenced by the internal clock of each listener. Previous studies have focused on “processing speed,” which has commonly been defined as the rate at which a task can be completed with reasonable accuracy (Jacobson et al., 2011). Although processing speed is similar to the idea of the internal clock, these two notions are conceptually distinct. Processing speed is associated with accuracy in the context of specific cognitive tasks, including memory tasks (Kail and Salthouse, 1994). If SMT reflects processing speed, participants who exhibit faster SMTs can be expected to recall the original music tempo more accurately than participants who exhibit slower SMTs, in which context the correlation between accuracy and SMT would be significant. To assess this possibility, we calculated the absolute difference between the presented music tempo and the adjusted music tempo in the recall task (8-s interval) and analysed the correlation between this difference and SMT; no significant correlation was observed in this context (*r* = 0.001, *p* = 0.995). On the basis of previous studies and the results of the current research, we conclude that SMT, which reflects individuals’ internal clock rather than processing speed, affects recalled music tempo following 8-s intervals.

Baudouin et al. (2006) conducted an experiment in which participants performed tasks that involved the production and reproduction of specific durations. As part of this time production task, participants were required to perform an action for a given duration that was defined in terms of regular time units (e.g., pressing a key for 30 s). In the time reproduction task, participants reproduced a previously presented target duration. The internal clock and processing speed exhibited by each participant were assessed. Their results suggested that time production was related to individuals’ internal clock, whereas time reproduction was related to processing speed. In the recall task employed in the current study, participants were required to reproduce a tempo after it was presented. From this perspective, the recall task used in the current study is analogous to the time reproduction task in the study by Baudouin et al. (2006). However, SMT, which reflects individuals’ internal clock, was related to performance in the context of memory task, a finding that was not replicated in the study conducted by Baudouin et al. (2006). One notable difference between their study and the current study lies in the stimuli used in these contexts: the former study used durations during which a visual stimulus was presented, whereas the current study focused on music tempo. In the present study, processing speed was not assessed; thus, the role played by processing speed in the recall task remains unknown. Nevertheless, our findings suggest that individuals’ internal clock may influence performance on the recall task (8-s interval). Music is a complex stimulus that involves elements such as rhythm, melody, and harmony (Scruton, 2011), all of which can affect perceived tempo (Hammerschmidt et al., 2021). Therefore, the reproduction of music tempo may require these additional musical elements to be considered, which could be related to individuals’ internal clock. In addition, other studies have suggested that the timing of single-interval durations and isochronous timing are supported by distinct mechanisms (Breska and Ivry, 2018; Grube et al., 2010; McAuley and Jones, 2003; Teki et al., 2011). Thus, the discrepancy observed between the results reported by Baudouin et al. (2006) and those of the present study may stem from differences in the underlying timing processes. Future researchers should investigate the roles played by the internal clock and processing speed in the reproduction of musical tempo in further detail; they should also explore the separate contributions of these factors to single-interval duration and isochronous timing.

In the present study, the tapping task was included at the end of the experiment to prevent the tapping tempo from influencing the recalled and preferred music tempos. The same piece of music was presented to all participants at the same initial tempo, and the order of presentation was randomized to minimize the potential impact of prior music tempos on finger tapping. Nevertheless, it remains possible that previous exposure to music tempo—including during the tempo adjustment phase—affected the subsequent finger tapping tempo. A previous study reported that finger tapping tempo can be influenced by prior exposure tempo (Aridan and Mukamel, 2016). Based on both the current study and previous studies, future researchers should assess the causal relationship between exposure to music tempo and finger tapping tempo. In terms of gender, the current study included only one female participant. While some studies have reported no gender differences in tempo preference (Karageorghis et al., 2006), others have identified gender-related effects in temporal reproduction tasks (Roeckelein, 1972; Strang et al., 1973; Rammsayer and Lustnauer, 1989; Wittmann and Szelag, 2003). To ensure the generalizability of the current findings, future studies should investigate whether the contribution of SMT differs by gender. One might argue that the fixed effect sizes identified in the current analysis are small and may have limited practical predictive value in isolation. Nevertheless, the fact that SMT can predict adjusted tempo provides valuable insights into the cognitive mechanisms underlying tempo recollection. Notably, the current model, which relies solely on SMT and original tempo, does not account for other potentially influences such as the number of notes or familiarity, both of which have been reported to significantly affect tempo adjustment in previous studies (Hine et al., 2022). Although these variables were not included in the present study, they are essential to the task of developing a prediction model that exhibits practical relevance. To improve the practical utility of such a model, future studies should incorporate a wider variety of musical stimuli and a more diverse participant sample, thereby enhancing their generalizability and the robustness of their predictions.

In conclusion, our study revealed that SMT, which reflects individuals’ internal clock, is related to recalled music tempo over an 8-s interval as well as to preferred music tempo, whereas SMT is not related to recalled music tempo in the absence of such an interval. These results suggest that the rehearsal of presented music tempo may be affected by individuals’ internal clock. Future studies should assess this possibility directly. Additionally, an investigation of how the internal clock and processing speed contribute to the retrieval of music tempo—and how they differentially affect single-interval duration and isochronous timing—could provide insights into how memory and preferences are constructed.

## Data Availability

The raw data supporting the conclusions of this article will be made available by the authors, without undue reservation.
